# Training-related changes in early visual processing of functionally illiterate adults: evidence from event-related brain potentials

**DOI:** 10.1186/1471-2202-14-154

**Published:** 2013-12-13

**Authors:** Melanie Boltzmann, Jascha Rüsseler

**Affiliations:** 1 Department of Experimental Psychology, University of Bamberg, Markusplatz 3, 96047 Bamberg, Germany

## Abstract

**Background:**

Event-related brain potentials (ERPs) were used to investigate training-related changes in fast visual word recognition of functionally illiterate adults. Analyses focused on the left-lateralized occipito-temporal N170, which represents the earliest processing of visual word forms. Event-related brain potentials were recorded from 20 functional illiterates receiving intensive literacy training for adults, 10 functional illiterates not participating in the training and 14 regular readers while they read words, pseudowords or viewed symbol strings. Subjects were required to press a button whenever a stimulus was immediately repeated.

**Results:**

Attending intensive literacy training was associated with improvements in reading and writing skills and with an increase of the word-related N170 amplitude. For untrained functional illiterates and regular readers no changes in literacy skills or N170 amplitude were observed.

**Conclusions:**

Results of the present study suggest that the word-related N170 can still be modulated in adulthood as a result of the improvements in literacy skills.

## Background

Visual stimuli evoke a negative-going wave peaking between 120 ms and 200 ms after stimulus onset, which is often referred to as N170. Although this component is triggered by any visual stimulus, the amplitude strongly depends on the perceptual familiarity of certain stimulus classes. Several studies report larger N170 amplitudes in adult readers to word-like stimuli compared to visual control stimuli such as symbol strings [1-3].

In a longitudinal study, Maurer and colleagues investigated the development of the word-related N170 by comparing words and symbol strings in pre-literate children, literate children and adults [3-5]. In pre-literate children, the N170 did not differentiate between words and symbols [3], but was higher for words compared to symbols after the first two years of formal reading education [4]. Investigations of the same children in the fifth grade [5] and the comparison between children and adults [4,5] showed that the N170 specialization for printed words continues to develop with further reading experience. In skilled adult readers, the N170 was reduced compared to second grade children, suggesting a u-shaped, non-linear development of the N170 specialization for printed words. Adults and fifth graders also have shorter N170 latencies and stronger left lateralization than second grade children [5].

The findings regarding the visual recognition of pseudowords are not consistent. While some studies found no differences between words and pseudowords [1,3], other studies reported higher amplitudes for pseudowords relative to words [6] or higher amplitudes for words relative to pseudowords [7]. These differential findings likely result from the large variability of tasks and different subject groups used across studies.

Brem et al. [8] demonstrated that a brief training of grapheme-phoneme correspondence in pre-literate children results in an initial specialization for print. Before training, words and symbols activated similar bilateral regions in the posterior occipito-temporal cortex. Afterwards, words elicited higher activations than symbols, mainly in the left occipito-temporal cortex [8]. These findings can be interpreted as evidence for a premature recognition of words that already exists in early childhood. Yoncheva et al. [9] examined the impact of a short-term training with an artificial script on the N170 in adults. They trained two groups of normal reading adults to associate visual characters with corresponding spoken words. One group was instructed to associate embedded letter-like figures with phonemes (grapheme-phoneme group), while the other group was instructed to focus on the whole visual character (whole-word group). After the training, visual characters produced a left-lateralized N170 in the grapheme-phoneme group and a right-lateralized N170 in the whole-word group. The results were interpreted as evidence for the phonological mapping hypothesis, which states that the left-lateralization of the word-related N170 to trained visual words is associated with the extent to which students focus on grapheme-phoneme-associations [9].

The N170 amplitude is also increased for other familiar stimuli like faces [10] or for visual classes for which some individuals have developed a special expertise (e.g. birds [11], cars [12], or fingerprints [13]). Training with novel objects (“greebles”) also leads to an increase of the corresponding N170 [14]. Altogether, these results suggest that visual experience with certain classes of visual stimuli lead to fast specialized processing of these visual stimuli. However, faces and other visual objects produce a bilateral or right-lateralized N170 [10,15], whereas the word-related N170 is lateralized to the left [1-3].

The source of the word-related N170 is supposed to coincide with a particular area located in the mid-portion of the left fusiform gyrus [9,16], the putative visual word form area (VWFA, [17,18]). This assumption is supported by evidence showing that the N170 is generated in left ventral occipito-temporal regions, as demonstrated by intracranial recordings [19,20] and source localization estimates of electroencephalographic [3,5,15,21] and magnetencephalographic recordings [22]. However, the proposal of a visual word form area in the left ventral occipito-temporal region is controversial [23,24]. There is evidence that the VWFA is not only devoted to the visual processing of letter strings [17], but is also associated with naming, viewing or generating verbs [25-27]. Additionally, the VWFA is also activated when words are presented in the tactile (i.e. in Braille [28,29]) or auditory modality [30]. These findings show that the left ventral occipito-temporal region is a polymodal area showing considerable functional heterogeneity.

Regardless of the underlying brain structures, the N170 reflects the earliest consistent processing of linguistic stimuli and is used to investigate fast recognition processes in good as well as poor readers. A number of studies provide evidence that the fast visual specialization for print is reduced in dyslexic adults [31,32] and children [21]. *Developmental dyslexia* is an unexpected difficulty with accurate and fluent reading which occurs despite normal intelligence, motivation and exposure to adequate reading instruction and in the absence of sensory, psychiatric and neurological disorders. The failure to develop fluent reading skills often persists into adolescence and adulthood, while reading accuracy improves over time. Neurobiologically, dyslexic children and adults show reduced activity in two left posterior brain systems, one parieto-temporal and one occipito-temporal [33].

Functionally illiterate adults are characterized by even greater difficulties during reading acquisition than dyslexic individuals. The term *functional illiteracy* refers to adults who have attended school for several years but who failed to acquire functional reading skills. Although they received instructions in reading and writing, they left school with literacy skills that are at least three to four years below the expected level of performance [34]. As a result, they can use written language only to a very limited extent; they are unable to read and understand even short sentences [35]. A recent survey concludes that there are about 7.5 million functional illiterates in Germany (14.5% of the adult population [36]). Similar prevalence rates are reported for other industrialized countries, e.g. 9% for France [37] or 16% for the United Kingdom [38]. For the United States it is estimated that about 43% of the adult population are not able to use printed materials for everyday activities in an appropriate way [39]. Functional illiteracy is often associated with specific personal obstacles in childhood concerning school (e.g. truancy, inappropriate instructions, repetition of classes) and family environment (e.g. neglect, drug abuse of parents, abuse, numerous siblings etc.). However, these negative experiences do not apply for all individuals with low literacy skills, and are also not sufficient to let someone become functional illiterate [35]. Accordingly, some researchers propose that functional illiteracy results from cognitive deficits coupled with environmental disadvantages [35,40,41].

In the present study, we investigated visual word recognition in functionally illiterate adults. Participants were considered as functionally illiterate when the literacy skills were below the expected level of an average student in fourth grade (see Methods section for further details). Previous studies have demonstrated that visual word recognition, reflected by the N170, is strongly dependent on literacy skills and develops when children learn to read [4,5]. We used an implicit reading task to explore whether the N170 of functionally illiterate adults changes as a result of literacy training. We therefore compared N170 amplitude in a group of functional illiterates with a group of normal reading adults before and after literacy training. We expected that functional illiterates show a distinct N170 for words and symbols since they attended school for several years and therefore have some knowledge in reading and writing. However, the word-related N170 was expected to be smaller than the one of normal readers because of the difference in reading skills.

Functional illiterates have difficulties reading even simple words. Therefore, they probably decode words and pseudowords in a similar way. Consequently, we assumed that words and pseudowords produce a similar N170. Normal readers, on the other hand, read real words easily due to the high familiarity. Since pseudowords are unknown to them, the N170 should be smaller for pseudowords than for real words. For symbols, no differences between functionally illiterate adults and normal reading adults were expected. Moreover, the N170 as response to words and pseudowords should be lateralized to the left, while the N170 for symbols should be similar in both hemispheres or right-lateralized.

We further investigated whether the N170 can still be modulated in adulthood. To answer this question, a group of functional illiterates participated in an intensive literacy training. The N170 for word-like stimuli (words and pseudowords) should increase during the training, whereas the N170 for symbols is not supposed to change. For the group of normal readers, no changes in fast visual recognition processes were expected.

Finally, we compared training-related changes in literacy skills and N170 amplitude between functional illiterate adults taking part in different literacy trainings differing mainly in the intensity of the delivered training. The experimental group participated in a high-intensity training (trained group) and the control group participated in a low-intensity training (untrained group).

## Results

### Training and testing

Table 1 presents descriptive characteristics of each group for the variables sex, age, handedness and non-verbal IQ.

**Table 1:** Characteristics of participants: sex and handedness as well as means and standard deviations of age and general cognitive ability for each group

	**Functional illiterates**		**Regularly reading**
	**Trained (*n*** **= 20)**	**Untrained (*n*** **= 10)**	**Controls (*n*** **= 14)**
Sex	13 male	7 male	3 male
Handedness	16 right handed	10 right handed	13 right handed
Age	41.20 (9.59)	42.00 (11.27)	34.21 (10.94)
Non-verbal IQ	86.70 (7.41)	90.10 (12.22)	104.29 (14.58)

Overall, participants had an average non-verbal IQ of 93 (*SD* = 14) with scores ranging from 73 to 127. While the IQ scores of trained and untrained functional illiterates were comparable, both groups differed significantly from regular readers (trained group: *T*(32) = -4.63, *p* < .001; untrained group: *T*(22) = -2.51, *p* < .05). For sex, age and handedness no differences between both groups of functionally illiterates were found. However, the experimental group differed from the normal reading control group in sex ratio (*χ*^*2*^(1) = 7.82, *p* < .01). Handedness on the other hand was similar in both groups (*χ*^*2*^(1) = 0.93, *p* = .34), while age differences marginally missed significance (*T*(32) = 1.97, *p* = .06). The illiterate control group differed from the normal reading control group in sex ratio (*χ*^*2*^(1) = 7.08, *p* < .01), but not in handedness (*χ*^*2*^(1) = .0.8, *p* = .37) and age (*T*(22) = 1.70, *p* = 0.10).

Reading and writing abilities of both functionally illiterate groups before and after the training were analyzed using a two-way, repeated measures ANOVA with two levels of SESSION (pretest vs. posttest) and two levels of GROUP (trained vs. untrained group).

**Figure 1:**
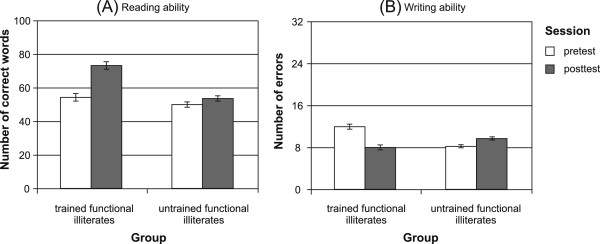
Reading (A) and writing (B) ability of trained and untrained functional illiterates at pre- and posttest.

For *reading ability*, the ANOVA revealed a significant main effect of SESSION (*F*(1,28) = 11.39, *p* < .01) and a significant interaction of SESSION by GROUP (*F*(1,28) = 5.26, *p* < .05). According to post-hoc paired *T-*tests, only trained functional illiterates improved in their reading ability from pre- to posttest (*T*(19) = -4.27, *p* < .001), while untrained functional illiterates did not show any considerable changes in their reading scores (Figure 1-A). Furthermore, trained and untrained functional illiterates did not differ in their reading ability at pretest (*T*(28) = -0.36, *p* = .72).

For *writing ability*, a significant interaction of SESSION by GROUP was found (*F*(1,28) = 15.17, *p* < .001). This effect is mainly due to the fact that trained functional illiterates significantly reduced the number of errors in the standardized writing test (*T*(19) = 4.27, *p* < .001), while there were no statistically reliable changes in the group of untrained functional illiterates (Figure 1-B). Importantly, trained and untrained functional illiterates did not differ in their writing ability prior to training (*T*(28) = 1.03, *p* = .31).

Reading and writing abilities of regular readers were assessed with normative tests appropriate for adults without reading difficulties (reading ability: SLS 5–8; writing ability: RT). All subjects scored within a range that can be expected for their age and formal educational status.

### Behavioral data

Results of the behavioral task (accuracy, number of false alarms and response time) are presented in Table 2, separately for each group.

**Table 2:** Means and standard deviations for behavioral data

			**Functional illiterates**		**Regularly reading controls (*n*** **= 14)**
			**Trained (*n*** **= 17)**	**Untrained (*n*** **= 10)**	
Accuracy in %	T1	Symbols	70.59 (19.44)	77.73 (14.55)	66.15 (25.01)
		Words	88.53 (13.08)	90.45 (9.61)	98.40 (4.00)
		Pseudo-words	82.94 (11.60)	86.67 (10.72)	97.12 (3.95)
	T2	Symbols	80.59 (8.99)	78.18 (19.01)	75.00 (17.08)
		Words	95.59 (6.09)	90.50 (11.17)	99.36 (2.31)
		Pseudo-words	87.94 (10.62)	87.92 (11.69)	96.79 (4.22)
Number of	T1	Symbols	4.35 (6.15)	7.18 (6.76)	3.23 (3.24)
False alarms		Words	0.24 (0.66)	0.73 (1.10)	0.31 (0.63)
		Pseudo-words	065 (0.93)	1.60 (2.12)	0.38 (0.65)
	T2	Symbols	6.53 (7.48)	5.91 (6.96)	3.69 (7.18)
		Words	0.47 (1.01)	0.60 (0.84)	0.00 (0.00)
		Pseudo-words	0.76 (1.30)	0.90 (0.88)	0.23 (0.44)
Response time in ms	T1	Symbols	10 (74)	642 (41)	648 (58)
		Words	630 (63)	631 (69)	635 (30)
		Pseudo-words	614 (71)	624 (48)	634 (36)
	T2	Symbols	623 68)	645 (35)	641 (48)
		Words	644 (82)	643 (40)	657 (47)
		Pseudo-words	647 (84)	651 (31)	651 (49)

Three-way ANOVAs with CONDITION (words vs. pseudowords vs. symbols), SESSION (pre- vs. posttest) and GROUP (trained group vs. untrained group vs. regular readers) were calculated for each measure of behavioral performance.

The ANOVA conducted for *accuracy* revealed a main effect of CONDITION (*F*(2,76) = 63.12, *p* < .001), SESSION (*F*(1,38) = 7.62, *p* < .01) and a significant interaction of CONDITION by GROUP (*F*(4,76) = 5.74, *p* = .001). The interaction between CONDITION and GROUP was significant for words vs. symbols (*F*(2,38) = 4.05, *p* < .05), pseudowords vs. symbols (*F*(2,38) = 8.32, *p* < .01), and words vs. pseudowords (*F*(1,38) = 3.29, *p* < .05). The superior performance for word-like stimuli compared with symbols was more pronounced in the normal reading control group than in both groups of functional illiterates (all *T* > 2.08, all *p* < .05). Functional illiterates were more accurate in detecting words compared to pseudowords, while normal reading controls detected words and pseudowords with similar accuracies (all *T* > 2.39, all *p* < .05).

Regarding the *number of false alarms*, results showed a significant effect of CONDITION (*F*(2,76) = 31.80, *p* < .001). There were more false alarms in the symbol than in the word (*F*(1,38) = 35.77, *p* < .001) and pseudoword condition (*F*(1,38) = 28.51, *p* < .001). Subjects also made more false alarms when they had to detect pseudowords compared to words (*F*(1,38 = 10.32, *p* < .01).

For the *response times* to correct identifications, no significant effects were observed.

### EEG data

**Figure 2:**
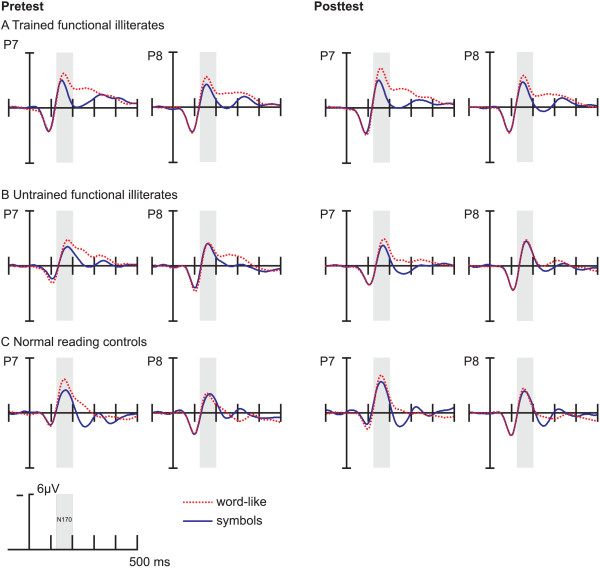
**Grand average ERPs time-locked to the onset of stimuli at occipito-temporal (P7/P8) electrodes for pre- (left) and posttest (right): (A) trained functional illiterates, (B) untrained functional illiterates and (C) regular readers.** Negativity is plotted up and each tick mark represents 100 ms of activity.

**Figure 3:**
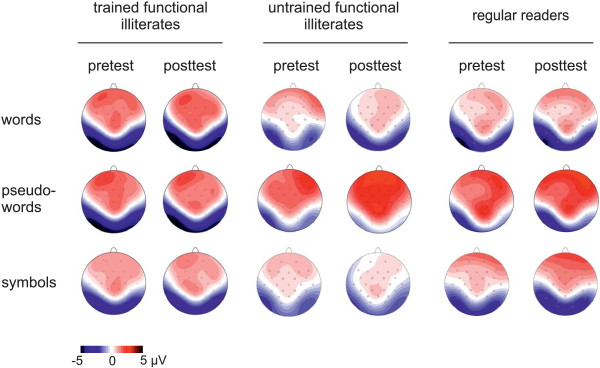
Spline-interpolated topographical maps for words, pseudowords and symbols at pre- (T1) and posttest (T2) are shown (time window: 120 to 200 ms).

In all groups, words, pseudowords and symbol strings elicited a posterior N170 between 120 and 200 ms at left (P7) and right (P8) occipito-temporal electrodes (see Figure 2, for corresponding topographical maps see Figure 3).

#### Peak latency

The average peak latency of the N170 was 161.29 ms (*SD* = 16.15 ms) across conditions, sessions, electrodes and groups. A four-way ANOVA with CONDITION (words vs. pseudowords vs. symbols), SESSION (pre- vs. posttest), HEMISPHERE (left vs. right) and GROUP (trained group vs. untrained group vs. regular readers) conducted for P7/P8 revealed no statistically reliable differences between these peak latencies.

#### Peak amplitude

First, we tested for differences in amplitude of the word and pseudoword-conditions. The two conditions did not differ in component structure or amplitude (see Figure 2). Thus, for all subsequent analyses, we averaged words and pseudo-words resulting in a new stimulus condition termed word-like.

We then tested if there were group differences at pretest. The results showed that the peak amplitudes of the N170 did not differ between the groups across electrodes and conditions (all *T* < 1.84, all *p* > .05). The peak amplitudes of the N170 were entered into a repeated measures ANOVA with the within subject factors CONDITION (word-like vs. symbols) and SESSION (pre- vs. posttest) and the between subject factor GROUP (trained group vs. untrained group vs. regular readers). The ANOVA was conducted for the left occipito-temporal electrode P7. Results showed a significant effect of CONDITION (*F*(1,41) = 26.81, *p* < .001) and a significant interaction between CONDITION, SESSION and GROUP (*F*(2,41) = 4.86, *p* < .05). Post-hoc *T-*tests revealed that the N170 was higher for word-like stimuli compared to symbols (*T*(43) = -5.78, p < .001).

**Figure 4:**
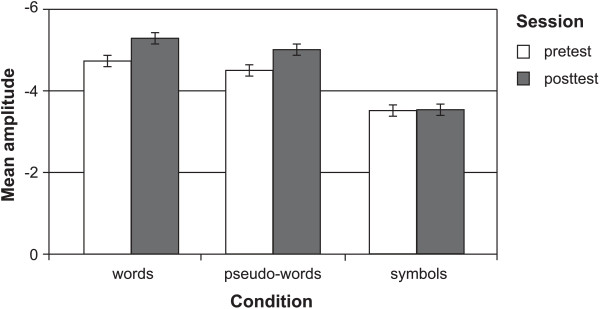
Mean amplitudes of words, pseudowords and symbols for pre- and posttest at the left occipito-temporal electrode (P7), for trained functional illiterates.

Because the three-way ANOVA evidenced a significant interaction of CONDITION by SESSION by GROUP, this effect was tested separately for each group with two-way ANOVAs (CONDITION x SESSION). For *trained functional illiterates*, the analysis revealed a main effect of CONDITION (*F*(1,19) = 21.27, *p* < .001) and a significant interaction between CONDITION and SESSION (*F*(1,19) = 9.12, *p* < .01). These effects reflect enhancements of the left N170 for word-like stimuli (pre: *M* = -4.61, *SD* = 2.42; post: *M* = -5.14, *SD* = 2.92; pre- vs. post-training at P7: *T*(19) = 2.17, p < .05), while the left N170 for symbols did not change over time (pre: *M* = -3.51, *SD* = 2.07; post: *M* = -3.52, *SD* = 2.57; *T*(19) = 0.02, p = 0.96), as depicted in Figure 4.

*Untrained functional illiterates* displayed only a significant main effect of CONDITION (*F*(1,9) = 9.12, *p* < .01), due to a slightly higher N170 for word-like stimuli than for symbol strings (*T*(9) = -3.76, p < .01). The peak amplitudes for word-like stimuli (pre: *M* = -3.57, *SD* = 1.62; post: *M* = -3.40, *SD* = 1.54; *T*(9) = -1.30, p = .23) and symbol strings (pre: *M* = -2.95, *SD* = 1.40; post: *M* = -2.61, *SD* = 1.42; *T*(9) = -1.05, p = .32) did not change from pre- to posttest.

For *regular readers*, results showed a significant main effect of CONDITION (*F*(1,13) = 6.63, *p* < .05), which was caused by larger amplitudes for word-like stimuli compared to symbols (*T*(13) = -2.58, p < .05). The ANOVA also revealed a trend for the factor SESSION (*F*(1,13) = 4.39; *p* = .06). According to post-hoc *T-*tests, the N170 for symbols (pre: *M* = -3.35, *SD* = 3.19; post: *M* = -4.13, *SD* = 2.68; *T*(13) = 2.42, *p* < .05) increased from pre- to posttest, while no significant changes were found for word-like stimuli (pre: *M* = -4.65, *SD* = 3.07; post: *M* = -4.99, *SD* = 2.82; *T*(13) = 1.25, *p* = .23).

**Figure 5:**
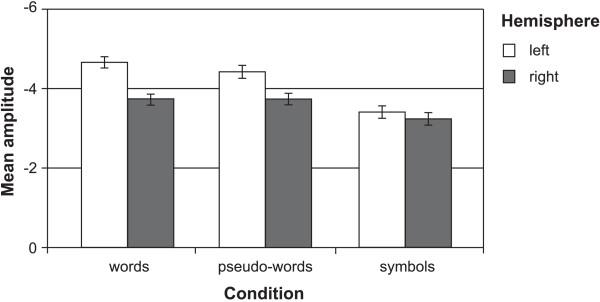
Mean amplitudes of words, pseudowords and symbols for left and right hemisphere.

To test for lateralization differences between P7 and P8, a four-way ANOVA with the additional within-subject factor HEMISPHERE (left vs. right) was computed. This analysis revealed a significant effect of CONDITION (*F*(1,41) = 30.34, *p* < .001) and a significant interaction between CONDITION and HEMISPHERE (*F*(1,41) = 9.03, *p* < .01). According to post hoc *T-*tests, the N170 amplitude was left-lateralized for word-like stimuli (*T*(43) = -2.49, *p* < .05), while the N170 amplitudes for symbol strings were similar in both hemispheres (*T*(43) = -0.60, *p* = .55), as can be seen in Figure 5.

#### Correlations between ERP data and reading ability

The difference in N170 amplitude between word-like stimuli and symbol strings at P7 was computed and correlated with the reading ability (number of words read correctly on the WLLP) at pre- and posttest. For *trained functional illiterates*, the increase of the N170-difference from pre- to posttest correlated positively with reading ability at pretest (*r* = 0.58, *p* < .01), and at posttest (*r* = 0.44, *p* = .054). Additionally, the N170-difference between words and symbols correlated with reading ability at pretest (*r* = 0.84, *p* < .001) and at posttest (r = 0.52, p < .05).

Moreover, participants with higher amplitudes of the word-related N170 at pre- (*r* = .55, p < .05) and posttest (*r* = .53, p < .05) showed larger improvements in reading. These correlations were still significant after controlling for subject’s age and cognitive ability. No significant correlations were observed for *untrained functional illiterates*.

#### Correlations between ERP data and behavioral performance

Behavioral analyses revealed that subjects were more accurate in detecting words and pseudowords compared to symbols. Therefore, we examined whether the different task difficulty is reflected in the ERP data, i.e. correlates with N170 amplitudes. As a result, behavioral performances in the different conditions did not correlate with the corresponding N170 amplitudes (all *r* < -0.21, all *p* > .16).

## Discussion

The present study examined visual word recognition of functionally illiterate adults. The word-related N170 is an ERP component which reflects fast recognition of visually presented words, and develops within the first years of reading acquisition [4]. We investigated with an implicit reading task whether the N170 can still be modulated when adults learn to read fluently.

In accordance with previous findings [1-3], the present study found a larger N170 for word-like stimuli than for symbol strings. In contrast to our initial hypothesis, functionally illiterate adults also showed a distinct N170 for word-like stimuli and symbols, which resembled the clear N170-difference of normal reading adults. Additionally, the N170 amplitudes were left-lateralized for words and pseudowords, which is in line with other studies [1-3]. Brem and colleagues demonstrated that children initially use a broad bilateral network in the ventral posterior occipito-temporal cortex when they have to recognize words [8]. With increasing reading expertise this bilateral network is progressively confined to the left hemisphere [4]. Accordingly, the literacy skills of functional illiterates obtained in childhood seem to be sufficient to account for the left-lateralized discrimination between word-like stimuli and symbol strings.

Behaviorally, all groups were more accurate in detecting words and pseudowords compared to symbol strings. The reduced accuracy and the increased number of false alarms in the symbol condition suggest that the task was especially difficult to perform for this stimulus type. Memorizing symbol strings, which were equally unfamiliar to the participants, seems to be more difficult than memorizing words and pseudowords [42]. However, these behavioral differences were detected in all groups, and did not correlate with the corresponding N170 amplitudes. Moreover, similar effects of stimulus type on accuracy were observed in previous studies using the same implicit reading task [3,7,16].

Functional illiterates detected repetitions of words and pseudowords less accurately than normal readers, which can be attributed to their different literacy skills. However, functional illiterates were more accurate in detecting words compared to pseudowords, indicating that detecting pseudowords was slightly more difficult for them compared to words. Maurer and colleagues propose an explanation in terms of the degree to which grapheme-to-phoneme conversion strategies are automatized. If the strategies are less automatized, subjects might use them to a lesser degree in implicit reading tasks [4]. Although functional illiterates probably have knowledge of the grapheme-to-phoneme rules, they are likely less developed than the grapheme-to-phoneme conversion skills of normal reading adults. This could explain why normal reading adults show no difference in detecting words and pseudowords, while functional illiterates are more accurate in detecting words compared to pseudowords.

As an important result of the present study, the amplitude of the word-related N170 has increased in functional illiterates after several months of intensive literacy training, whereas no changes occurred in the symbol condition. Consequently, improvements in literacy skills influence the word-related N170, even when adults learn to read fluently. The assumption that changes in the N170 are related to literacy skills is supported by a positive correlation between the enhancement of the word-related N170 and participant’s reading skills before and after training, evident only in the group of trained functional illiterates. In addition, the pre-test reading ability predicted the response to intervention: Higher reading abilities at the beginning of the training are linked to larger increases of the N170-difference and higher gains in literacy skills. A parallel fMRI study revealed that subjects who participated in the intensive literacy training showed significant increases of activity in the fusiform gyrus, including the visual word form area, after eight months of training [43]. Although it is difficult to localize the sources of a certain ERP component, several converging studies indicate that the word-related N170 is generated in the left fusiform gyrus [3,5,15,19-22]. Therefore, both EEG and fMRI evidence training-related changes in the visual word recognition of functionally illiterate adults, associated with the visual word form system in the occipito-temporal region.

In contrast to the experimental group receiving intensive literacy training, no changes were observed in functional illiterates receiving less intensive training. Although this group attended literacy courses as well, the present study demonstrated that literacy trainings held with a low frequency (only once a week for 1.5 hours) did not cause changes in literacy skills and in early visual word recognition.

In normal reading adults, the word-related N170 also did not change over time. This result was very much expected, because skilled readers differ from functionally illiterate adults in their proficient literacy skills. Hence, they process words on a very high level reflected by stable N170 amplitudes at both test times. Surprisingly, the N170 for symbol strings seems to increase from pre- to posttest in the normal reading control group, as can be seen in Figure 2. Although the effect does not approach significance in the conducted ANOVA, post-hoc tests show a marginally significant increase from pre- to posttest for symbols only. As described in the Methods section, the normal reading control group consists of Psychology students as well as non-students, suggesting that this group differs from functionally illiterate adults in more aspects than literacy skills. The cognitive ability seems to be the most relevant factor, since members of the normal reading control group have higher non-verbal IQ values than both groups of functionally illiterate adults. The sub-group of students seems to particularly contribute to these differences. Therefore, we analyzed the data reported in the Result section again excluding students this time. The results did not change with regard to the word N170, as the amplitudes were still comparable at both test times. Interestingly, there was a considerable change in the N170 elicited by symbols. At pretest, the symbol N170 remained similar to that obtained before with students included, but at posttest the symbol N170 was significantly reduced. It can therefore be concluded that the increase of the symbol N170 from pre- to posttest was caused by the inclusion of students.

We further examined whether cognitive ability and age were related to changes in N170 amplitudes across all subjects. Although age had no impact on N170 changes from pre- to posttest, a significant correlation between cognitive ability and the increase of the N170 for symbols was found: subjects with a higher cognitive ability demonstrated a greater increase of the N170 for symbols. This could be attributed to the fact that cognitive ability was measured with the Culture Fair Test (CFT). In this test, subjects have to figure out relations between shapes and figures. Similar symbolic material is used in the CFT and the paradigm of the present study which might explain the positive correlation between these variables. Since students have a higher cognitive ability than the other participants, this could explain the greater increase in the symbol N170. Considering these findings, a control group more similar to functionally illiterate adults is needed in future studies. Additionally, it should be further investigated how the symbol N170 is modulated by cognitive ability.

Brem and colleagues examined changes of the N170 for words and symbol strings in adults when stimuli are presented repeatedly. The N170 for symbols changed in amplitude and topography, whereas the N170 for words remained constant [2]. Since those changes only occurred for symbols, they cannot solely be attributed to the repeated presentation of stimuli. They rather reflect the development of a visual competence for symbols. It is likely that Psychology students who might deal more often with symbolic material than the other participants developed a visual competence for symbol strings after single presentation.

Given the fact that functional illiterates do not show a reduced word-specific N170 compared to regular readers, the question arises why one would expect training-related changes in the N170 for words even though functional illiterates already reached the same level as proficient readers. This result might be more comprehensible in light of Maurer and colleagues’ longitudinal data regarding the development of N170 specialization. These studies revealed that in non-reading preschoolers, the N170 does not differentiate between words and symbols. During the first years of literacy instruction the N170 is increasingly tuned for words. Subsequently, the word-specific N170 decreases again in adolescence and adulthood when individuals become proficient readers [3,4]. In terms of the present results, we assume that functional illiterates classify between nonreading children in preschool and elementary school children. It might be reasonable that their N170 specialization has not yet reached its peak, but will still increase with additional reading practice and subsequently decrease when a certain literacy level has been achieved. Consequently, the N170 of functional illiterates and regular readers observed in the present study reflect different stages of N170 development. While functional illiterates probably still demonstrate increases with gains in literacy skills, it is not likely that regular readers show any changes in their word-specific N170 due to their long standing reading experience.

### Evaluation of the training

The experimental group who received intensive literacy training significantly improved their literacy skills and demonstrated word-related changes in N170 amplitudes. Previous studies confirm that adults with severe reading impairments can improve their reading skills ([44-46], for a review [47]). However, several recent intervention studies revealed only low to moderate effects in adults [48-50]). Children who were taught with similar approaches showed significantly larger improvements than adults. It appears that interventions that have proven to be effective in childhood are not easily transferable to the field of adult education. Especially the intensity of intervention programs is critical to their effectiveness. The present study can be seen as evidence that intensive literacy trainings are more effective than less intensive literacy trainings.

The two programs also differed in their methodological approaches. The depicted conventional courses rely on similar teaching strategies used during literacy education of children. Since these strategies already proved to be insufficient to teach functionally illiterate adults how to read and write in childhood, they were complemented with additional methods in the training for the experimental group. These methods comprised a training of perceptual abilities, audio-visual integration, and phoneme discrimination as well as joint social activities to strengthen self-confidence. However, the design of the present study does not allow to draw conclusions about the effectiveness of the individual methods implemented in the current program. Further research is required to investigate whether the employed methods have differential effects on the training success. In future studies, literacy trainings have to be offered with the same intensity if the effectiveness of alternative approaches is evaluated.

## Conclusions

Functionally illiterate adults showed a larger N170 response to words and pseudowords than to symbol strings, indicating a visual expertise for linguistic stimuli. A group of functionally illiterate adults receiving intensive literacy training demonstrated improvements in their literacy skills which were linked to an increase of the word-related N170. In contrast, another group of functional illiterates attending less intensive literacy courses did not show any changes in the word-related N170. Our study demonstrates that the N170 can still be modulated in adulthood, when functionally illiterate adults learn to read fluently. However, intensive literacy training is necessary to induce training-related changes.

## Methods

### Participants

44 healthy adults participated in the present study: 20 functional illiterates who took part in AlphaPlus (*trained group*), 10 functional illiterates who received a conventional training (*untrained group*), and 14 non-impaired readers who received no training (*normal readers*).

The group of 20 trained functional illiterates participated in a newly developed reading and writing training program (13 male; mean age: 41.20 ± 9.59 years, aged 25-52 years). All subjects were recruited by collaborators of local job centers and participated voluntarily. Dropout had no financial consequences for the participants, i.e. was not sanctioned by cutting social security benefits. Subjects attended school (primary and secondary school) between six and twelve years (*M* = 9.35 ± 1.27 years). Only two participants had completed vocational training. At the time of the course, all participants were unemployed. The members of the trained group did not attend any other literacy courses for adults prior to their participation in the present course.

The group of untrained functional illiterate adults comprised 10 individuals who did not participate in the new training program (7 male; age: *M* = 42.00, *SD* = 11.27, range: 29-67 years). Instead, they attended conventional literacy classes at adult education centers; at the time of the study for about 3 years (*M* = 3.06, *SD* = 2.70 years). Courses were usually held once a week for one and a half hour per session. Subjects of this group attended school for about 8 years (*M* = 7.7, *SD* = 2.58 years). Three of the ten participants had completed vocational training. At the time of the study five participants were unemployed, four employed and one was retired.

The group of regular reading adults consisted of 14 non-impaired adult readers (3 male; age: *M* = 34.21, *SD* = 10.94, range: 20-50 years) who responded to newspaper advertisements and other postings. Subjects of this group attended school for 8 to 12 years (*M* = 10.71, *SD =* 1.44 years). Six subjects were unemployed, two employed and six were students (B.Sc. Psychology).

All participants were native German speakers with normal or corrected-to-normal vision. According to a structured interview, none of them had a history of neurological or psychiatric disorders in child- or adulthood. Moreover, none of them was officially diagnosed as dyslexic in childhood. The study was conducted in accordance with the declaration of Helsinki. Furthermore, the study protocol was approved by the ethical committee of the University of Bamberg. All participants gave their written informed consent and were paid for test participation (10 € per hour).

Data of 16 additional subjects (trained group: *n* = 6, untrained group: *n* = 6, regular readers: *n* = 4) were not included in the analyses because of too many artifacts in EEG-data (*n* = 14) or technical problems (*n* = 2).

### Stimuli and procedure

Subjects participated in two sessions, which were on average seven months apart (trained group: *M* = 7.40, *SD* = 0.34; untrained group: *M* = 7.41, *SD* = 0.26; regular readers: *M* = 7.46, *SD* = 0.24). Participants were tested during the first four weeks after the training started and again four weeks before the program officially ended. Recordings took place in a quiet room with subjects seated in front of a computer screen.

**Figure 6:**
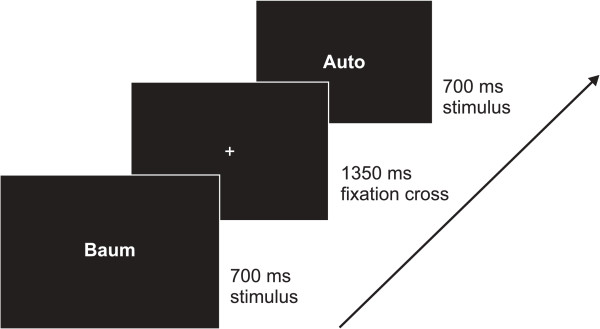
**Illustration of the paradigm for the word condition.** “Auto” is the German word for “car”; “Baum” is the German word for “tree”.

The procedure of the present study was adapted from Maurer and colleagues (e.g. [3,4]). The stimuli of all conditions (symbol strings, words, pseudowords, pictures) were shown in black on a white background in the center of the screen. Each condition consisted of 120 stimuli, which were divided into two blocks of 60 stimuli each. The stimuli contained 20% immediate repetitions serving as targets, with the second stimulus being excluded from ERP analysis. Subjects were asked to press a mouse button with their index finger whenever a stimulus was immediately repeated. The behavioral task was applied to maintain subjects’ attention at the center of the screen. Each stimulus was presented for 700 ms followed by an interstimulus interval (ISI) of 1350 ms. During the ISI, a black fixation cross was presented on the screen (see Figure 6).

Symbol strings, words, and pseudowords were matched for character size and number of characters in a string (3 to 6 characters). The word stimuli were concrete nouns derived from schoolbooks for first-graders (e.g. Baum *(engl. tree)*). The word stimuli had an average word frequency in the CELEX database (occurrence per 6 million [51]) of 292.91 (*SD* = 631.48), ranging between 2 and 5174.

Pseudowords were pronounceable nonsense words (i.e., words without meaning) corresponding to the phonological and orthographical rules of German (e.g. Gabo). The first letter of each word and pseudoword was capitalized followed by letters written in lowercase. Symbol strings were randomly assembled from a set of ten different geometrical symbols (). Stimuli for the picture condition were line drawings derived from the Snodgrass pictures; 88 from the standardized set developed by Snodgrass and Vanderwart [52] and 12 from the extension presented by Cycowicz et al. [53]. The sequence of stimuli and blocks were pseudorandomized for each participant. Stimuli were presented with the software *Presentation* (Neurobehavioral Systems Inc.; http://www.neurobs.com).

### EEG Recording and data analysis

The electroencephalogram was recorded from 27 sintered Ag-AgCl electrodes integrated in an elastic cap (positions: Fp1/Fp2, F3/F4, C3/C4, P3/P4, O1/O2, T7/T8, P7/P8, Cz, Fz, Pz, Fc1/Fc2, Cp1/Cp2, Po3/Po4, Fc5/Fc6, Cp5/Cp6). Electrodes were positioned according to the standard 10/20 system [54]. A quickamp amplifier (Brain Products GmbH, Munich, Germany; http://www.brainproducts.com) was used for the EEG registration. Horizontal and vertical electrooculograms (EOG) were recorded to control for eye movement artifacts. Blinks and vertical eye movements were monitored with electrodes affixed above and below the left eye, whereas lateral eye movements were recorded by two electrodes placed on the left and right external canthus. Scalp electrodes were referenced against average reference. Electrode impedances were kept below 5 kΩ. The electrophysiological signals were amplified with a bandpass of 0.01-70 Hz and a notch filter centered at 50 Hz, digitized online with 4 ms resolution (sampling rate of 250 Hz) and stored on hard disc.

From the continuous signal, stimulus-locked epochs of 1024 ms starting 100 ms prior to stimulus onset were averaged for each condition. The epochs were monitored offline for ocular and other artifacts by using individual thresholds. Subjects were excluded from analyses if more than 30% of trials in at least one condition had to be rejected. Event-related brain potentials were derived by averaging the remaining artifact-free epochs per condition. A pre-stimulus period of 100 ms served as baseline for ERP computation.

ERP data were quantified by peak amplitude measures at lateral occipito-temporal scalp sites (P7/P8) taken between 120 ms and 200 ms (N170). We selected occipito-temporal electrodes as the N170 has previously shown to be maximal at these channels (e.g. [2,21]). Furthermore, the peak latency was identified for each condition, session and electrode of interest.

Based on the results of Maurer and colleagues [3,4] we compared the processing of words, pseudowords and symbol strings to investigate whether there is a specialization for linguistic stimuli in functionally illiterate adults and to detect training-related changes. Data of the picture stimuli are not reported, which is consistent with most studies of Maurer and colleagues [4,7,21].

The ERP data were submitted to a repeated measures ANOVA with the within subject factors CONDITION (words vs. pseudowords vs. symbols) and SESSION (pre- vs. posttest) and the between subject factor GROUP (trained group vs. untrained group vs. regular readers). To test for lateralization differences, a four-way ANOVA with the additional within-subject factor HEMISPHERE (left vs. right) was computed.

Follow-up ANOVAs and post hoc *t* tests were performed to specify observed effects. The uncorrected degrees of freedom and the corrected p-values are reported.

### Reading and writing instructions

Members of the experimental group participated in a literacy training, specifically developed for functionally illiterate adult (Alpha plus). Functional illiterates are often ashamed of their deficits and constantly afraid of public humiliation. Consequently, they run a high risk of social withdrawal. Therefore, the main objective of the training program was to improve the literacy skills in a way that the participants would be able to self-organize their daily life and overcome their social isolation.

The training program was implemented in two courses that were offered over a period of nine months, i.e. from March until November 2009 (course 1) and 2010 (course 2), respectively. Professional tutors conducted the program in a local educational institute in northern Germany. Lessons took place from Monday to Friday between 8.30 am and 2 pm. The training consisted of three main modules with different methodological foci. First, the basic rules of mapping graphemes to phonemes were taught in conventional literacy lessons (approx. 2 hrs. daily). This approach is similar to instructions given to children during regular literacy acquisition. Specific paper-pencil based exercises were developed due to the lack of appropriate educational materials for adult basic education. The training material was highly relevant to the everyday issues of the participants in order to achieve sustained success of the training and to ensure that the participants will engage in reading and writing in their everyday life. The training began with reading of syllables; later, short words and sentences were used. One of the main objectives was that participants learn the rules of mapping graphemes to phonemes and internalize the phonological structure of words. It was important to ensure that the participants really read the words by phoneme-grapheme-conversion and not just by pure memorization. Common German letter combinations were trained intensively.

The second module was characterized by the use of technical devices to train different skills relevant for language processing (approx. 2 hrs. daily). This part of the training was supposed to consolidate the achievements made during conventional reading lessons. Among other things, the participants trained with a tool called *BrainBoy*® to improve their basic visual and auditory perceptual abilities. In eight subtests, participants have to discriminate different features of tones or light flashes (for more information see http://www.meditech.de). In addition, audio-visual integration processes were trained with a tool called *AlphaTrainer*. Linguistic stimuli like words are visually shown on a computer screen and simultaneously vocalized by a speaker, whose voice is presented via headphones. The participants themselves have to vocalize the presented stimuli into a microphone. The two language streams (speaker’s voice and participants’ voice) are distinctly perceived either with the left or with the right ear, and they change in opposite directions (e.g. speaker’s voice from left to right, own voice from right to left). The input sides of the two language streams constantly change after a few seconds. It is assumed that the synchronous reading of letters and the vocalizing of words facilitates the audio-visual integration of letters and speech sounds. This idea is based on findings showing that dyslexics have deficits in audio-visual integration [55]. Moreover, training of audio-visual integration has previously been shown to improve literacy skills in dyslexic children [56,57].

Finally, the discrimination of phonemes or consonants with similar sound structures was trained. Via headphones, three-letter pseudowords were presented. All of them had as first letter an “e” and as last letter an “i”. The central character was always a consonant, which varied from trial to trial (e.g. “ebi”, “eki”, “epi”). The participants were asked to type the perceived middle character on a computer keyboard.

Social activities like shopping in the supermarket, cooking, visiting the stadium of the local soccer team or fire department were also part of the program. This social training finally constitutes the third module of the program.

### Cognitive testing

The Culture Fair Test (CFT, [58]) was used to assess overall intelligence. The CFT is supposed to measure fluid intelligence independent of verbal fluency, cultural background, and educational level. The four subtests (series, classifications, matrices, conditions) are non-verbal and require participants to figure out relationships between shapes and figures. In the present study, scale 2 (German version) was used because it is applicable for adults with basic education. Only form A was applied in order to reduce the test time. Subjects were included in the study if they had an IQ of 70 or above.

Reading ability of functionally illiterate adults was assessed with the WLLP (*Würzburger Leise-Leseprobe*, [59]). In this test, 140 written words as well as four pictures next to each word are presented. Participants have to mark the one picture that represents the word on the left side. The test score comprises the number of correctly identified pictures in five minutes. The WLLP is supposed to measure silent reading speed and the ability to decode written words. A standardized German test for the first grade was used to assess the writing ability of functional illiterates (*Diagnostischer Rechtschreibtest*, DRT 1, [60]). Here, participants have to write 32 single words from dictation. We used parallel versions of the WLLP and DRT 1 at the beginning as well as at the end of the training in order to examine whether the reading and writing abilities had changed over time. Since there are no specific criteria defining functional illiteracy, the present study refers to different national studies that used a norm-oriented approach. In these studies, subjects were classified as functionally illiterate when their performance did not meet at least the performance of an average student of the fourth class. To assess whether individuals meet this criterion, the WLLP was used since it allows a comparison of individual scores with students in first to fourth grade. However, all of our participants were at or below the average reading level of a student in the middle of the second grade.

For the regular readers, normative tests appropriate for adults were used to evaluate their individual literacy levels. These tests also have been applied to identify subjects suffering from reading and/or writing disabilities, e.g. dyslexia. Affected subjects were excluded from the study. Tests developed for first grade students were not a valid option for this group because they are neither sensitive nor specific for adults. We selected the SLS 5–8 (*Salzburger Lesescreening für die Klassenstufen 5–8*, [61]) to test the reading abilities and the RT (*Rechtschreibungstest*, [62]) for the writing abilities.

## Competing interests

The authors declare that they have no competing financial or non-financial interest in the research reported here.

## Authors’ contributions

MB and JR designed the study, prepared the stimulus material and wrote the manuscript. MB collected and analyzed the data. Both authors read and approved the final manuscript.
